# Pedestrian Navigation Based on a Waist-Worn Inertial Sensor

**DOI:** 10.3390/s120810536

**Published:** 2012-08-03

**Authors:** Juan Carlos Alvarez, Diego Alvarez, Antonio López, Rafael C. González

**Affiliations:** Multisensor Systems & Robotics Lab (SiMuR), Department of Electrical and Computer Engineering, University of Oviedo, Campus de Gijón, Edificio n°2, Gijón 33204, Spain; E-Mails: dalvarez@uniovi.es (D.A.); amlopez@uniovi.es (A.L.); corsino@uniovi.es (R.C.G.)

**Keywords:** pedestrian dead-reckoning, inertial navigation, localization, location based services, ambulatory monitoring, human motion

## Abstract

We present a waist-worn personal navigation system based on inertial measurement units. The device makes use of the human bipedal pattern to reduce position errors. We describe improved algorithms, based on detailed description of the heel strike biomechanics and its translation to accelerations of the body waist to estimate the periods of zero velocity, the step length, and the heading estimation. The experimental results show that we are able to support pedestrian navigation with the high-resolution positioning required for most applications.

## 1. Introduction

We present a personal navigation system (PNS) based on inertial measurement units (IMUs). A PNS is a device that computes its own position in indoor or outdoor terrains without depending on external signals. Our system consists of a commercial off-the-shelf IMU placed on the back of the user, near the body center of gravity (COG), and wirelessly connected to a handheld processing unit. The IMU has two functions: it measures gait-corrected inertial displacements and it detects periods of zero velocity, needed to increase the positioning resolution by correcting the IMU measures.

This device belongs to the class of the Pedestrian Dead Reckoning navigation systems (PDRs), which make use of the human bipedal pattern to reduce position error. Human bipedal gait consists of two phases: swing and stance [1]. The swing phase extends from the instant the toe leaves the ground until the heel strikes. These events are called Final Contact (FC) and Initial Contact (IC) respectively, and between them the foot is off the ground. The stance phase begins when the heel first contacts the ground, and extends while the foot rolls and it reaches the midstance, producing the forward motion of the body by pivoting of the leg on the ankle. During the midstance the vertical component of the velocity of the waist is zero, and this fact can be used to initialize the integration of accelerations, so diminishing drifts and reducing the position error (the zero velocity update strategy, ZUPT).

In order to better detect the step impact shock, most PDRs tend to place the IMU near the ground, usually on the heel and at the sole of the boot of the user. This detection technique typically results in a 1%–2% positioning error [2], enough for most indoor applications. Our objective was to design, build and test a waist-worn PDR system that could achieve similar resolution. Although foot mounted IMU locations have some advantages to implement the ZUPT strategy, the waist or trunk locations are probably the least intrusive IMU placement, are easier to wear and more convenient in some applications.

In this paper we describe improved algorithms to accurately estimate the periods of zero velocity, the step length and the heading estimation, based on detailed description of the heel strike biomechanics and its translation to accelerations of the body COG. The results show that we are able to detect zero-velocity points accurately enough to implement a PDR system worn on the user's waist, and to support pedestrian navigation with the high-resolution positioning required for most applications, close to a relative error about 2% of the distance traveled [3], and 8% of the turn angle [4], in indoor environments.

The remainder of the paper is set out as follows: Section 2 provides a literature review identifying related PDR systems and their characteristics. Section 3 outlines the ZUPT, the step length estimation and the heading estimation methods. Section 4 describes the PDR overall system and the experimental results to validate it. Finally, Section 5 discusses these results, identifies implications for future research and summarizes the paper.

## 2. Sensor Location Alternatives

Personal dead-reckoning systems have two components: a step detection subsystem with a pedometer-like function, and a direction subsystem to estimate the orientation. In this work we are not concerned with a possible third component to measure altitude changes such as elevators or stairs between different floors. Implementation can be made by attaching inertial sensors to the body to measure the patterns that are typical of the cyclical characteristics of human walking motion.

For example, the number of steps can be counted from accelerometers, such as in a common pedometer. A rate gyroscope accounts for orientation, by integration the rate of change with time, and initializing it with a GNSS or a magnetic compass if required. It is also possible to estimate step lengths in real time from the accelerometer signals. A calibration process is usually needed to compensate the individual variability of acceleration profiles.

Accumulative drifting errors are inherent to all these estimations, as they are based on adding noisy signals. In PDRs this problem is corrected by taking advantage of the cyclical nature of human walking: when the foot is on the ground, the velocities and accelerations of the shoe are zero, and it can be taken as the starting point of a new estimation, or zero-velocity updating (ZUPTing).

The efficacy of all these subsystems depends on which sensor is utilized and where it is located. The sensor may be mounted or attached at any convenient point on the user's body, as long as it can sense the harmonic motion accelerations associated to walking or running. Several IMU locations have already been tested, e.g., the waist, trunk, leg, foot or even the head.

A shoe-mounted IMU is the most frequent location in MEMS-based PDR systems [5]. Results may vary depending on the specific sensor set or the experimental conditions. Errors up to 20% of distance traveled are common in abrupt terrains [6,7]. In [8] they reported a maximum distance estimation error of 5.3% over a 30 meter course, with a tri-axial accelerometer and a single axis angular rate sensor on the shoe.

Similar results are reported in [9] with a two-axis magnetometer located on a shoe and a Kalman filter to reduce magnetic disturbances in indoor environments, or in [10] with a single axis angular rate sensor leading to errors of 4% in 120 m. Ojeda, Borenstein *et al.* [3,11] use a small six-degree-of-freedom IMU attached to a user's boot, with a ZUPT technique that produces a relative error about 2% of the distance traveled, independent of the gait or the speed of the user. More recent shoe-mounted PDRs reach similar levels of indoor precision, from 1.2% in 370 m walks [4] to 10% for longer paths [12]. These results are usually best-case scenarios. It is not easy to make systematic comparative studies of PDRs performance, as usually conditions and methods are difficult to reproduce fairly [13].

Shoe-based PDRs' limitations could be overcome by adding more sensors to the system, at the expense of complexity and cost. For that reason, in [14] they use radio frequency phase changes between a reference signal located in a waist pack, and from a transmitter located on each boot. In [15], ultrasonic sensors attached to boots help to measure the length of every stride in real time, leading to a maximum error of 5.4% in straight-line walking. In [16] two IMUs, one on each boot, are used with the idea of limiting the drift error growth with the stride length estimation at each foot. In [2] a high-resolution thin flexible ground reaction sensor cluster (GRSC) is added to the shoe-worn IMU, for more accurate determination of the zero-velocity point in the ZUPTing subsystem, reporting that errors decrease from 0.4% to 0.35% in half-hour experiments (1,200 m walks, 4 m errors) compared to gyroscope-based ZUPTing.

In this work we choose to locate the IMU at the body COG, as a waist-worn device. The reasons for this are: (1) shoe sensors may be impractical if they require shoe modifications or wires up to the leg of the user; (2) waist-worn sensors are less intrusive and more convenient in some applications because we are more accustomed to carrying some other devices on the belt; and (3) waist-worn IMUs have better results for heading estimation using gyroscopes or magnetometers [8].

Previous work on PDRs with waist-worn sensors have their roots in the work of Levi and Marshall [17] who developed the first commercial system. Step detection is made by processing the fundamental component of the vertical acceleration combined with peak detection of the signal. Step length is experimentally related to step frequency for each individual, and orientation is estimated from a magnetometer signal and individual calibration. Later the system is expanded to deal with lateral and backward displacements. Ladetto *et al.* [18] deal with step count by peak detection in the vertical and antero-posterior accelerations, and the step length is estimated from the step frequency. The system in [19] is similar but step length is estimated by a heuristic formula, individually calibrated, which leads to reported errors of 3% inter-individual and about 8% intra-individuals. Orientation is estimated by the combined use of accelerometers and gyroscope. Other works in the literature are based on post-processing of the sensor signals, and are not suitable for localization in real-time [20].

In this paper, we describe a PDR with an IMU located at the COG, and able to deliver state-of-the-art precision. For displacement estimation, it will be necessary to improve both the zero velocity detection and the step length estimation, by means of biomechanical models of walking. The results show that good precision can be achieved, and therefore it is not mandatory to renounce such an advantageous sensor position, so convenient for a lot of applications. Below, the sensors, each method used in the system and their integration approach is explained in detail.

## 3. Signal Processing Methods

### 3.1. Zero Velocity Detection Algorithm

Our goal is to detect the mid-stance event (MS), located between the events of Final Contact (FC) and Initial Contact (IC) that delimit the stance phase of walking (while one leg pivots in the air). In biomechanics, this event corresponds to when the swinging foot passes the reference or pivoting foot, and happens to coincide with the instant when the COG is in its highest vertical position. Also, this event is posterior but very close to the instant of flat foot.

In order to define an improved algorithm to accurately detect zero-velocity stance from the waist, we need a detailed description of the heel strike biomechanics and its translation to accelerations of the body COG. For shoe-worn PDRs, most stance-based schemes in the literature equate zero-velocity detection to the impact of the heel when it hits the ground, or Initial Contact (IC) event, which can be easily identified from the foot IMU acceleration sensor signals. Biomechanics studies show that this event coincides in time with when the body center of gravity (COG) is in its lowest vertical position, a more convenient description for waist-worn PDRs. Below, we describe an improved algorithm to accurately detect zero-velocity stance from the waist. The objective is to define the data needed to capture the nature of the contact with the ground, to accurately detect the periods of zero velocity.

**Figure 1.**
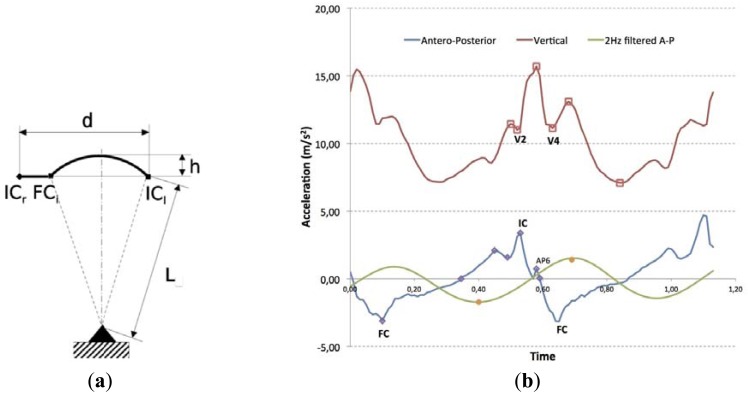
Gait events and their notation in vertical and antero-posterior trunk accelerations. (**a**) Step length estimation between Initial Contact events (IC) from left and right foot, using the Final Contact event (FC) to limit the integral; (**b**) IC and FC events reflection in measured trunk accelerations, and how they are detected in real-time.

Our algorithm has its roots in the description of gait events given by [21,22] from the vertical acceleration of the lumbar area. Such a description, see Figure 1(a), shows a multimodal signal for each step composed of three main peaks, where the initial contact corresponds to the first valley (V2) and the second valley (V4) is associated with the contralateral final contact. Regarding the antero-posterior acceleration, the higher peak (IC) corresponds with the Initial Contact, and the lower peak with the Final Contact (FC).

From this description, the first step for event detection is to compute the principal harmonic of the vertical acceleration using a 30 order, zero-lag, low-pass FIR filter with a cutoff frequency of 2.5 Hz. This filtered signal is used to locate the maximum of the vertical acceleration. ICs are marked at the maximum of the AP signal, immediately before a vertical acceleration maximum. Final contacts FCs are located as local minimum in a small neighborhood after each maximum in the vertical acceleration. Experiments show a number of false detections of six ICs and eleven FCs out of 4,675 steps [23]. Acceleration data was captured using an IMU placed close to the L3 vertebra with its measurement axes aligned to the anatomical ones, and secured through the use of a corset.

The previous algorithm needs some modifications in order to be implemented in real-time. To improve robustness to miss-detections and other signal artifacts, the search of peak V2 in the vertical acceleration is substituted by the location of zero crossings from positive to negative in the output of an 11th order FIR filter applied to incoming antero-posterior acceleration. Possible false zero crossings (not associated to a real IC event) are detected through the analysis of the positive lobe that precedes each found zero crossing, which must be almost constant for true zero crossings, and significantly greater for false zero crossings. A threshold is used to efficiently separate both kinds of lobes. Also, the search of the IC peak is limited to search windows heuristically defined. Experiments in real-time detection showed that accuracy for the IC event timing is 13 ± 35 ms; accuracy for FC events timing is of 9 ± 54 ms [24]. These results are sufficient to back up the idea that this algorithm is adequate for a waist-worn PDR system.

### 3.2. Step Length Estimation

The most direct way to estimate the length of step with inertial sensors is through the double integral of the signal from an accelerometer oriented in the direction of motion. However, it is known that errors of this method grow quadratic in time, so it becomes worthless in practice. A solution is to periodically reset the IMU drift by detecting the instants when the velocity is zero (ZPTUing). Thus, stride length is estimated by integrating the horizontal acceleration between successive foot contacts (IC), where the foot has zero velocity. Other alternatives to avoid the integration error such as to estimate the step length from the walking speed [25], are not effective under general conditions, because they depend on heuristics that must be particularized to the individual by methods of statistical adjustment in the initial calibration process, based on averages taken in periods of uniform walking speeds.

For IMUs worn at the waist, the length of step can be estimated by measuring the vertical displacement of the COG after double integration of vertical acceleration. This idea, originated in the scope of gait analysis, can also be applied for PDR systems [26]. This estimation is based on the assumption that the vertical movement of center of mass during a step, delimited by two consecutive IC events, is equal to that described by a point mass suspended at the end of an inverted pendulum. The step cycle is divided into two phases: double stance (from IC to contralateral Final Contact FC) and single stance (from contralateral FC to next IC), see Figure 1(b). The inverted pendulum model is the step length estimator during single stance, but during double stance displacement is considered constant and related to the foot size. The algorithm needs the subject's leg length measured from the external malleolus to trochanter major (L2), the subject's foot length (F) and the vertical acceleration signal of the step, from the previous IC event of the contralateral foot to the following IC event of the reference foot. Then, the antero-posterior displacement *d* can be related to vertical displacement according to equation:

(1) $$d=2\sqrt{2Lh-h^{2}}$$

being *L* the leg length, and *h* the vertical displacement of the COG during the single stance, computed by a double integration of the waist vertical acceleration. The zero velocity update (ZUPT) is made in the IC event, which defines the step start.

This method has been tested experimentally under laboratory conditions, walking in a straight line of about 20 m (19.92 ± 0.28 m), walking at constant speed. The reported precision is 8.8% with accuracy of 100.96% [27]. The record shows that it is a comparable alternative to other previous methods, suitable to be a component in the construction of a waist-worn PDR system.

### 3.3. Heading Estimation Using Gyroscopes

Walking direction can be computed by integrating the signal of a vertical gyroscope, or by direct measurement of magnetometers. Magnetometers produce absolute values that have a deviation due to the magnetic declination, and they are unreliable because of environmental magnetic disturbances. By numerical integration of a vertical gyroscope signal we can estimate orientation, but with drift errors that accumulate and grow unbounded in time. In this case, we have no biomechanical model to help us to implement a ZUPT strategy to avoid that.

**Figure 2.**
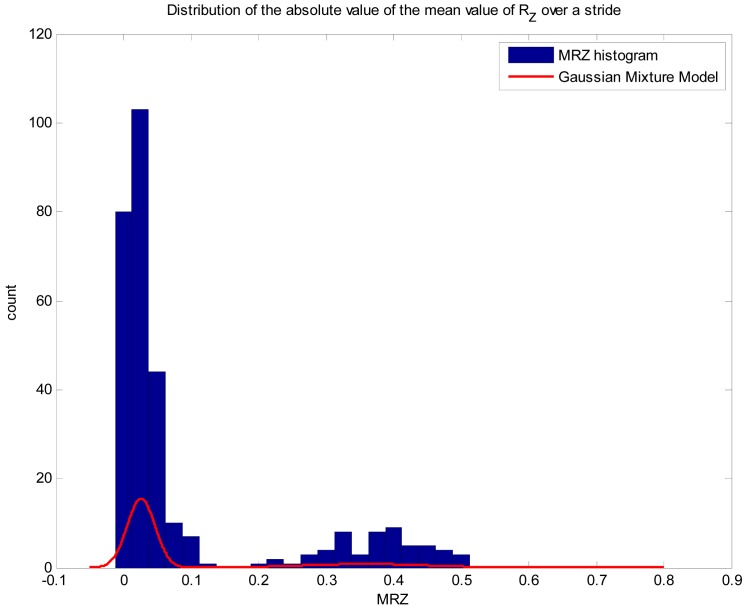
Distribution of the absolute value of the mean of R_z_ over a stride. The peak near to zero corresponds to straight walking, while the peak to the left corresponds to turning steps. The red line shows the fitted Gaussian mixture model.

**Figure 3.**
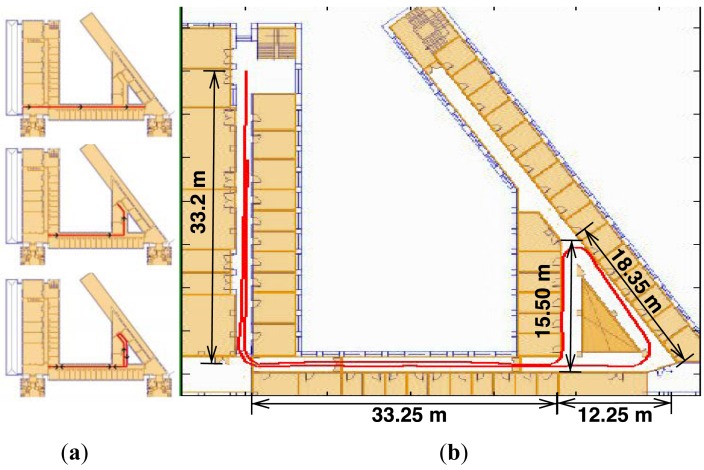
Location C for large-range indoor experiments, 179 m length with 540 degrees turns. (**a**) Partial shorter experiments within the same environment, see Table 1; (**b**) Real-time path reconstruction.

The approach we take to overcome this limitation has two parts. First, we implement a basic algorithm to distinguish whether the individual is moving in a straight line or is turning, in order to disable the gyro integration to avoid unnecessary error accumulation. We assume that rotation speed around the vertical axis (Z axis) has two components, an AC component with the same frequency as the stride, which accounts for trunk rotation during normal gait, and a lower frequency component associated to the orientation change rate. Under this assumption, the absolute value of the mean of the Z component of rotation speed over a stride (MRZ) should be close to zero for straight walking and greater than zero for curved walking. Taking the absolute value allows to get rid of rotation direction. Figure 2 shows the distribution of MRZ for walk experiments in location of Figure 3(a).

To solve the classification problem, we fit a Gaussian mixture model. We have used three independent experiments consisting in the completion of two rounds around the track shown in Figure 3(a). The first trial was used to fit the model, the second to validate it and the third to test the results. Fitted model is shown as a red line in Figure 2. The precision of the classifier is 1, so every step labeled as curved walking corresponds to actual curved walking. On the other hand, the value for the recall parameter is 93.65%, meaning that almost 7% of curved walking steps where labeled as straight walking. Those false negatives are related to very small curves (which take less than a stride). Every straight walking step was correctly classified. To solve the false negative problem we have decided to integrate two straight steps occurring before and after a curved walking step. This classification algorithm works in real-time and only requires storing the data of four steps.

Once a stride has been selected, we integrate the gyro signal to calculate the change in orientation, but experiments show that it is not enough by itself to produce angle estimations in the range needed. We increase accuracy by estimating bias through a double calibration, both for the user (user and sensor placement) and for the sensor itself. This calibration is quite simple and consists in completing a small path with two simple requirements: should we small (30 to 50 steps at user preferred speed) and should complete a 360 turn, *i.e.*, the user must finish facing the same initial direction. Using this information, we may estimate bias using the formula:

(2) $$b=\frac{\int_{0}^{T}\hat{w}(t)dt-2\pi }{T}$$

where *ŵ*(*t*) is the calibrated signal from the sensor (rad/s), 2π accounts for the total gyro and T is the duration of the test. This bias is used later for real-time compensation. The results show that the combination of both ideas is sufficient to produce orientation estimations useful within some range of conditions, but not good enough for long distance walks.

## 4. Experiments

Our PDR system is based on a sensor unit consisting of a tri-axial accelerometer and an uniaxial gyroscope placed in the lumbar area of the individual, in a position close to the COG. Starting from a known initial position, the device estimates the new position and orientation at each step. It is therefore necessary to detect the occurrence of the step with the minimum time delay possible. This is made with the detection algorithm described in the previous section, as well as the step length and orientation estimations.

Acceleration data was captured using an IMU placed close to the L3 vertebra with its measurement axes aligned to the anatomical ones, and secured through the use of a corset. Raw acceleration and gyro data were gathered by means of an Xsens MTx device. This sensor includes a triaxial accelerometer, a triaxial gyro and a triaxial magnetometer, being the total MTx size 40 × 55 × 22 mm. Acceleration measurement range is ±10 g, with a linearity error of 0.2% of FS, bias stability (1*σ*) 0.02 m/s^2^ and bandwidth 30 Hz. Angular velocity range is ±1,200 deg/s, with a linearity error of 0.1% of FS, bias stability (1*σ*)) 1 deg/s and bandwidth 40 Hz. Magnetic field measurement range is ±750 mGauss, with a linearity error of 0.2% of FS, bias stability (1*σ*)) 0.1/mGauss and bandwidth 10 Hz. Alignment error between the different sensor axes is less than 0.1 deg. A signal acquisition system, Xsens XbusMaster, transmits the measured signals via Bluetooth or USB to a PC laptop. Measurements were made with a group of eight adult volunteers, with ages 24 to 45, along none of them appearing to have any impairment in the locomotion system that could affect the experiments.

The sensor placed in the lumbar area of the pedestrian has to be aligned with its motion axis. Because the sensor is placed arbitrarily on the surface of the lumbar zone, the initial misalignment is corrected by an initial calibration procedure. From the initial signals acquired at rest, we calculated a correction matrix that will be used while in real-time operation. The sensor undergoes small misalignments during a step sequence because of the movement of the support lumbar surface. We have considered this factor negligible because of the low amplitude expected for the misalignment.

Indoor experiments were carried out at the Electrical and Computer Engineering Department building, in the Polytechnic School of Gijon. It is an office environment, with a Geographic Information System (gis.uniovi.es) that maps all university buildings geographically, and can also show the floor plans of each of them, providing accurate mapping information. This information was contrasted on the ground with a laser range finder, in the specific areas where the experiments were made.

**Figure 4.**
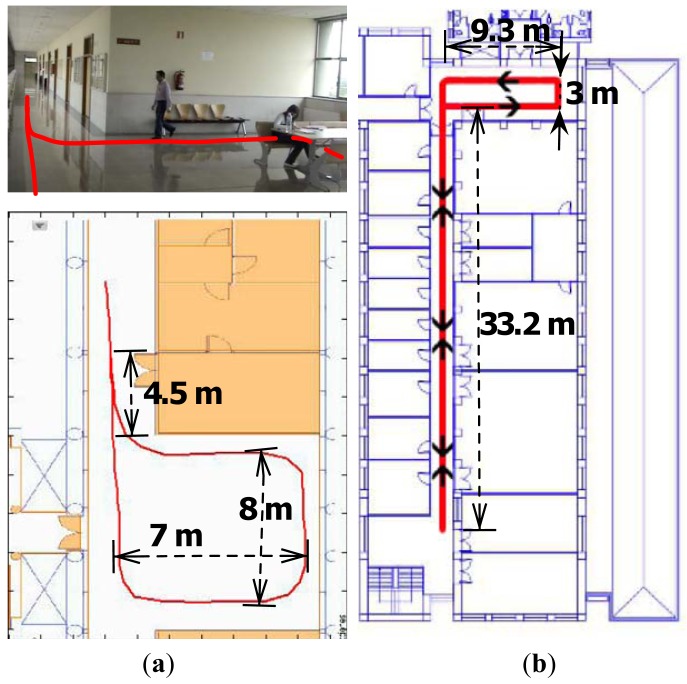
Short-range indoor experiments. (**a**) Location A, a closed path 39 meters long and 360 degrees turns, real-time results of one experiment; (**b**) Location B, a closed path 91 meters long with 360 degrees turns.

We performed short and long range experiments in three different locations. Initial walking direction is introduced by hand. The short distance runs were made in two office environments, represented in Figure 4(a). A 39 m long square was used for the first test and adjustment purposes. It has a total rotation of 360 degrees, 270 degrees to the left and 90 degrees to the right. The path ends at the same initial point, but with a 180 degree orientation with respect to the start. An extended version of these trials, but 91 m long, was made in Location B in Figure 4(b). Finally, Figure 3 depicts experiments in Location C, a 179 m long path with a total 360 degrees left turn and 180 degree right turn, that is, 540 degree overall. Trajectories in both Figures 3 and 4 are those produced in real-time by the developed PDR system.

Subjects were recruited from the pool of volunteers, in order to record four to six trials at each location, on different days. Trials were discarded when they produced unacceptable errors. That happened in approximately one out of six trials. By visual inspection of the discarded data, we have identified that there is a potential problem when the patterns of antero-posterior and vertical acceleration (Figure 1), are so irregular that the ZUPT method produced several erroneous detections. This is due to a variety of reasons that could range from the individual walking conditions (e.g., the specific shoes used, lateral steps, walking velocity, and so on), to unwanted sensor artifacts coming from skin and muscle movements on the waist during walking. More analysis is required to better understand these experimental limitations of the system. Even though, we already reported similar limitations in previous systematic experiments, 31 out of 192 trials, when walking in straight line under restricted lab conditions [27], and therefore the results reported here, under more realistic conditions, do not represent a significant worsening. The total walking distance is calculated by adding all the step length for each excursion. Similarly, turn angle is calculated by adding the estimated turn angle per stride.

**Figure 5.**
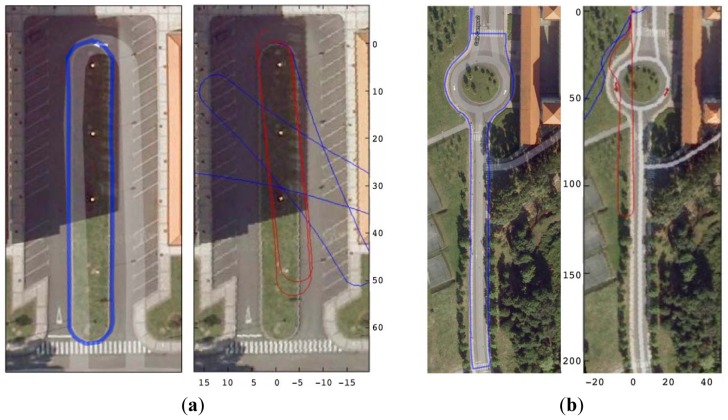
Orientation estimation outdoor tests, with uncalibrated step length. (**a**) Path 240 m long; (**b**) Path 460 m long (axis in meters). Tracks (**left**) and their corresponding real-time reconstruction (**right**) in two scenarios, by using only motion state detection (blue path), and by motion detection + calibration (red path).

Orientation estimation being the most difficult part, we carried out long distance outdoor walks to provide more insight into the performance of this component of the PDR. We tested the combination of individual calibration for users and tracks, and the motion detection idea to avoid unnecessary gyro integration. Figure 5 shows the better results obtained when adding calibration to motion detection. Experiments were made outside the department building, using a commercial GPS positioning system, over two predefined paths, of 460 m and 240 m respectively.

## 5. Results

A total of almost 2 km with 6,660 degrees of turns were recorded indoors. Results of the experiments are summarized in Table 1 for the three selected indoor locations. Four to six typical runs are selected for each location. The table includes the percent error between measured and estimated distance, at each location. The added median distance error was 2.1% in distance, with no apparent dependence with distance within the range of the experiments (40 to 180 m). Percent error in turn angle estimation is computed using *Err_θ_* = (*θ_real_* − *θ_est_*)/180, what leads to a 100% error when the estimation is opposite to the real orientation.

Turn errors are greater and more difficult to reduce because of the reasons previously exposed. In Figure 5 and Table 2 we can see the results of four different trials of Location C, with orientation errors under 2.3%, which makes the path still recognizable when plotted over the building planes. Trials where turn errors grew higher than 20% were discarded because they begin to be useless for localization and track reconstruction over a map.

Errors obtained in indoor scenarios (up to 180 m) for individual trials are under 5% in distance, and under 9% in final orientation, which can be considered a worst-case scenario. These numbers give us some hints about how to combine this PDR device with some kind of sensor network for localization, and what is the optimal distance between beacons that could help to complement the inertial device [19,28]. Then, a distance of 200 m between beacons could guarantee a bounded localization error of 5 m in distance.

The effect of orientation errors is more prominent in the long range outdoor experiments of Figure 5. The proposed algorithm combining calibration and conditional gyro integration gives acceptable results, but the variability of the results is greater, and the worst-case error grows to exceed the 15% in the tested scenarios. Outdoor walks were carried out on a flat pavement, so the effect of the irregular walking surface in city-like environments is not included in this study.

## 6. Conclusions

The proposed waist-worn PDR system delivers location information that can be precise enough for some applications. This information is provided in real-time and independently of any infrastructure, and not as the result of computer-based post-processing, making it useful for location-based applications and services. Walking orientation is by no means robust, and relays on pre-calibrated computations. Step count and step length estimation are much more reliable, and do not deteriorate a lot when increasing the walking distance. Experiments in city-like asphalt tracks show similar results. The experiments discarded because of failure of the ZUPT detection method represent another limitation. Further research is needed to identify the reasons for the occasional distortion of the acceleration signal at the waist, and to compensate them to avoid their negative effect, especially in the estimation of the turn angle orientation.

The measured error levels allow us to define the distance needed for a beacon-based infrastructure which could complement this PDR, in order to provide real-time pedestrian localization and tracking in GPS-denied environments, within the error margins demanded by the specific application.

Because the sensors are waist-worn, this work could be applied also to electronic devices we already carry with us, such as smart phones or music players. In this case calibration procedures are not valid anymore, as these devices are usually not fixed to the body but in the user pocket, and they move freely as we walk [29]. The three modules comprising the PDR should be revised taken this problem into account.

As conclusion, the development of more detailed algorithms for the inertial estimation of orientation shows the biggest potential to increase the PDR autonomy and its dependence on external aids. Ideas ranging from detecting variations in the magnetic field [30] to the use of biomechanical constraints [31] open the path to improvements, which could lead to provide authentic autonomous indoor positioning for a growing range of situations.

## Figures and Tables

**Table 1.** Indoor experiment results in Locations A, B, C in Figures 2 and 3. Real distance and turn degrees were measured at each location, and compared to estimated ones at the end of the trial.

**Location**		**Estimated Distance**		**Estimated Turn**	

**Id.**	**Real distance (m)/turn (deg)**	**m**	**%**	**Err. (deg)**	**%**

A	39/360	37.4	4.1	13.4	7.5

B	91/360	89.5	1.6	4.2	2.3

C	179/540	173.3	3.2	2.3	1.3
	54/0	54.3	0.6	8.9	5.0
	49.3/120	51.5	4.5	9.4	5.2
	100.6/420	97.7	2.9	16.2	9.0

TOT	1,925.6/6,660	1,884.7	2.1	80.5	6.2

**Table 2.** Detailed results of four trials in Location C, plotted in Figure 5.

**Location**		**Distance**		**Turn**	

**Id.**	**distance (m)/Turn (deg)**	**m**	**%**	**Err. (deg)**	**%**

C	179/540	173.48	3.1	3.13	1.7
		171.04	4.4	3.43	1.9
		172.06	3.9	4.09	2.3
		176.42	1.4	0.81	0.4
